# Role of Mycoplasma Chaperone DnaK in Cellular Transformation

**DOI:** 10.3390/ijms21041311

**Published:** 2020-02-15

**Authors:** Francesca Benedetti, Fiorenza Cocchi, Olga S. Latinovic, Sabrina Curreli, Selvi Krishnan, Arshi Munawwar, Robert C. Gallo, Davide Zella

**Affiliations:** 1 Institute of Human Virology, School of Medicine, University of Maryland, Baltimore, MD 21201, USA; fbenedetti@ihv.umaryland.edu (F.B.); fcocchi@ihv.umaryland.edu (F.C.); olatinovic@ihv.umaryland.edu (O.S.L.); SCurreli@ihv.umaryland.edu (S.C.); skrishnan@ihv.umaryland.edu (S.K.); amunawwar@ihv.umaryland.edu (A.M.); rgallo@ihv.umaryland.edu (R.C.G.); 2 Department of Biochemistry and Molecular Biology, School of Medicine, University of Maryland, Baltimore, MD 21201, USA; 3 Department of Medicine, School of Medicine, University of Maryland, Baltimore, MD 21201, USA; 4 Department of Microbiology and Immunology, School of Medicine, University of Maryland, Baltimore, MD 21201, USA

**Keywords:** Mycoplasma, DnaK, cancer, p53, microbiota

## Abstract

Studies of the human microbiome have elucidated an array of complex interactions between prokaryotes and their hosts. However, precise bacterial pathogen–cancer relationships remain largely elusive, although several bacteria, particularly those establishing persistent intra-cellular infections, like mycoplasmas, can alter host cell cycles, affect apoptotic pathways, and stimulate the production of inflammatory substances linked to DNA damage, thus potentially promoting abnormal cell growth and transformation. Consistent with this idea, in vivo experiments in several chemically induced or genetically deficient mouse models showed that germ-free conditions reduce colonic tumor formation. We demonstrate that mycoplasma DnaK, a chaperone protein belonging to the Heath shock protein (Hsp)-70 family, binds Poly-(ADP-ribose) Polymerase (PARP)-1, a protein that plays a critical role in the pathways involved in recognition of DNA damage and repair, and reduces its catalytic activity. It also binds USP10, a key p53 regulator, reducing p53 stability and anti-cancer functions. Finally, we showed that bystander, uninfected cells take up exogenous DnaK—suggesting a possible paracrine function in promoting cellular transformation, over and above direct mycoplasma infection. We propose that mycoplasmas, and perhaps certain other bacteria with closely related DnaK, may have oncogenic activity, mediated through the inhibition of DNA repair and p53 functions, and may be involved in the initiation of some cancers but not necessarily involved nor necessarily even be present in later stages.

## 1. Mycoplasma

Mycoplasmas are the smallest and simplest self-replicating bacteria, ranging from 0.1 to 0.3 μm in diameter and up to 200 μm in length. The mycoplasma cell contains the essential organelles needed for growth and replication: plasma membrane, ribosomes, and a genome consisting of a double-stranded circular DNA molecule. Unlike all other prokaryotes, the mycoplasmas have no cell walls, and they are consequently placed in the Mollicutes class (mollis= soft; cutis= skin) [1]. The plasma membrane is composed of approximately two-thirds protein and one-third lipid. Membrane proteins, glycolipids and lipoglycans exposed on the cell surface are the major antigenic determinants in mycoplasmas [2,3,4,5].

Mycoplasma species are widespread in nature: they are parasites of humans, animals, plants and arthropods. They usually exhibit organ and tissue specificity, and their primary localizations are the mucous surface of the respiratory and urogenital tracts, the eyes, the alimentary canal, the mammary glands and the joints [6]. Besides residing in contact with the cellular membrane, some mycoplasma species can invade the cells and become intracellular residents [7,8]. Because mycoplasmas lack cell walls, they can invade the host cells by interacting with the membrane of the host and fuse together the two membranes. Membrane fusion alters the composition and permeability of the host cell membrane and enable the introduction of the mycoplasma’s hydrolytic enzymes into the host cell, causing severe damages [9,10].

Although some mycoplasmas belong to the normal microbiota, many species are pathogens that can cause various diseases either asymptomatic or chronic. Seven species of mycoplasma (M. *pneumoniae*, M. *urealyticum*, M. *genitalium*, M. *hominis*, M. *fermentans*, M. *penetrans* and M. *pirum*) are mainly related to human pathologies, such as acute respiratory illness, genitourinary tract infections, joint infections and neurologic disorders [11,12,13,14,15,16]. In fact, mycoplasmas can elicit strong host immune responses due to the presence of lipoproteins on their membrane able to trigger a Toll-like receptor-mediated response, activate macrophages, induce cytokine production and lymphocyte proliferation [17,18,19,20]. Furthermore, because of the presence of antigenic variability systems, mycoplasmas are able to rapidly change the composition of the major surface protein antigens and consequently escape recognition by the immune mechanisms of the host [21,22,23].

## 2. Mycoplasma and COPD

Chronic Obstructive Pulmonary Disease (COPD) is the third leading cause of death in the United States, with more than 130,000 deaths annually. Worldwide, more than 3 million people die annually of COPD, mostly in middle- and low-income regions. Almost 15 million people have been diagnosed with COPD in the US alone, but millions more may have the disease without even knowing it. COPD causes serious long-term disability and early death. At this point, there is no cure, and the number of people dying from COPD is growing. The main cause of COPD in developed countries is smoking [24], while in developing countries COPD is also frequently seen in patients exposed to environmental air-polluting agents (for example burning fuel for cooking). COPD is characterized by chronic inflammation leading to progressive lung damage, which ultimately contributes to irreversible airflow obstruction [25]. An acute exacerbation of COPD is defined as increased shortness of breath and sputum production, a change in the color of the sputum from clear to green or yellow, or an increase in cough. Furthermore, the oxidant burden in the lungs of COPD patients, caused by an imbalance between the generation of free radicals and the antioxidant defense systems, increases the release of multiple inflammatory mediators with further reduction in responsiveness during anti-inflammatory treatments [26]. Dysbiosis is observed in lungs of patients of COPD, in that the composition of lungs microbiota is less diverse than the one observed in healthy persons, and has a tendency to be restricted to phyla comprising potentially pathogenic microorganism, including M. *pneumoniae* [24,27,28], also associated with acute exacerbation [29,30]. Some of the mechanisms by which M. *pneumoniae* could enhance the negative effects of tobacco products in COPD patients is by increasing oxidative stress [31], inflammation status and hypoxia-related factors, such as HIF-1α [32]. Quitting smoking in combination with anti-muscarinic bronchodilators resulted in an improvement of lung function and respiratory symptoms in mild COPD patients and it could be interesting to determine whether this would also result in a reduction in dysbiosis and the presence of M. *pneumoniae* [24]. An increased risk of developing lung cancer was observed in smokers with airflow obstructions, particularly of squamous histological subtype in patients diagnosed with non-small cell lung carcinoma, compared to smokers with normal lung functions [33,34,35], and it has been shown a prognostic effect of hypoxia (measured through expression of HIF-1α) with COPD and lung cancer [36].

## 3. Mycoplasma and Cancer

Mycoplasmas are part of the human microbiota: they are commensals, but they have also been associated with tumorigenesis. The correlation between mycoplasmas and cancer remains unclear [37,38], but epidemiological studies, in vitro experiments and genome sequence analysis indicate a close involvement of mycoplasmas in cellular transformation and cancer progression [39].

Several studies in vitro using bronchial epithelial cells [40], hepatocytes [41], oral tissues [42], human prostate cells [43,44] and cervical cells [45] indicate that the presence of mycoplasma may facilitate tumorigenesis by promoting cellular transformation [46,47]. In addition, experimental data indicates that mycoplasmas infection cause chromosomal changes, instability and cell transformations in vitro through progressive chromosomal loss and translocations [45,48,49,50]. It has been demonstrated that M. *fermentans*, M. *penetrans* and M. *hyorhinis* are able to induce the accumulation of chromosomal abnormalities and also phenotypic changes of the transformed cells [45,49,50]. In more detail—during the long-term infection of mouse embryo fibroblasts with M. *fermentans* or M. *penetrans* spontaneous cellular transformation and overexpression of the H-Ras and c-myc proto-oncogenes have been shown [51]. Infection with M. *fermentans*, *arginini* and *hominis* reduced the activity of p53 and induced the constitutive activation of the NF-κB transcription factor in mouse 3T3 cells [52]. The incorrect regulation of NF-κB has been in fact linked to cancer, inflammatory and autoimmune diseases, viral infection, and improper immune development [17,53,54,55]. Aberrant DNA methylation has also been observed in cancer [56]. M. *hyorhinis* encodes DNA-(cytosine-5)-methyltransferase enzymes that target CpG dinucleotides, establishing the methylation patterns of the bacterial genomes [57]. When expressed in human cells, they are able to translocate into the nucleus and create abnormal methylation patterns of the host cell DNA [57,58,59,60,61]. These epigenetics changes can contribute and lead to cancer progression by stimulating pro-oncogenic pathways. M. *fermentans*, M. *hominis* and M. *penetrans* have also been shown to cause the transformation of human lung cells and mouse myeloid cells and fibroblasts by inducing the bone morphogenetic protein 2 (BMP-2) [62], that is considered a marker for lung cancer when highly expressed and is also associated with poor patient survival [63].

More recently, it has been shown that M. *hyorhinis* induces the motility of gastric cancer cells by activating the Wnt/βcatenin signaling pathway through the membrane protein p37 [64], that has also been shown to significantly correlate with high vascular invasion and associated with poor disease-free survival of Hepatocellular carcinoma patients. In vitro studies suggest that M *hyorhinis* infection promotes tumor progression in HCC patients, by increasing the migratory capacity of HCC cells, through the interaction of p37 with epithelial cell adhesion molecule (EpCAM) [65]. The p37 protein alone may be considered sufficient to increase invasiveness and metastases of different cancer cells [41,66,67].

Many in vitro studies support the ability of some mycoplasmas to induce cancer transformation and many pro-cancer mechanisms have been investigated in the last years: from the induction of genetic instability [48,49], to alterations in metabolism [68] and changes in the expression of tumor suppressor or oncogenes [46,51,52,69]. However, no carcinogenic roles for any mycoplasma have been demonstrated in vivo. Several specimens from patients (infectious tissues, neoplastic tissues and body fluids) have been analyzed and mycoplasma has been isolated and detected in those samples without any formal demonstration of causality. In particular, mycoplasmas have been detected so far in many tumor tissues (precancerous lesions and malignant tissues) from patients with gastric, esophageal, colon, lung, breast, glioma, renal, ovarian, cervical and prostate cancers [67,70,71,72,73].

## 4. Bacterial DnaK

To cope with different stressful conditions and maintain the proper proteostasis, eukaryotic organisms have a redundant system of chaperone proteins [74]. One of these families (Hsp70) is comprised of slightly different proteins [75], and the over-expression of some members of the Hsp70 family increases the transformation of several human cell types [76,77]. On the other hand, suppression of Hsp70 expression by anti-sense Hsp70 cDNA inhibits tumor cell proliferation and induces apoptosis [78]. Though with some differences in amino acid sequences among the several bacteria species, the bacterial chaperone system is mainly organized around the DnaK protein (corresponding to the eukaryotic Hsp70), thus representing a central hub in prokaryotic protein interaction networks [79].

Chaperone activity of Hsp70/DnaK is controlled by cycles of ATP binding and hydrolysis [80]. DnaK itself is a weak ATPase, while direct interaction with the co-chaperone DNAJ proteins (members of the HSP40 family) [81,82] greatly increases ATPase activity, promotes binding with target proteins and accelerates protein-folding activity of Hsp70/DnaK [83]. There are three types of co-chaperone proteins comprising the DNAJ family: DNAJA (type I), DNAJB (type II) and DNAJC (type III) based on the presence or absence of conserved domains defined by the canonical DNAJ of E. *coli* [84]. It has been shown that specific DNAJ may play an important role in influencing cancer development and spread, possibly due to their role as co-chaperones influencing folding of various oncogenes or tumor suppressors [85,86,87,88,89,90,91,92,93,94,95,96,97,98,99,100,101,102].

## 5. Mycoplasma DnaK Interferes with Important Anti-cancer Cellular Pathways and Is Taken up by Bystander Cells.

We isolated and characterized a strain of human M. *fermentans* (MF-I) able to induce lymphoma in a Severe Combined Immuno-Deficient (SCID) mouse model [103,104,105], similar to a previously described lymphomagenesis dependent upon reduced p53 activity [106]. Mycoplasma was abundantly detected early in infected mice, but only low copy numbers of mycoplasma DnaK DNA sequences were found in primary and secondary tumors, suggesting a “hit and run/hide” mechanism of transformation, in which the critical events have occurred previous to cancer detection [107]. We demonstrated that this mycoplasma’s DnaK binds to human USP10 (ubiquitin carboxyl-terminal hydrolase 10, a regulator of p53 stability), reducing p53 stability and anti-cancer functions, potentially increasing the likelihood of DNA mutations and consequent malignant transformation [107]. P53 is a major tumor suppressor, often called “the guardian of the genome” for its multiple anti-oncogenic activities. By tightly coordinating cell cycle and apoptotic responses, p53 ensures that DNA damage is properly repaired, or that the damaged cell is removed upon engagement of the apoptotic pathway. P53 is mutated in about 50% of human cancers [108,109,110], and a mutated p53 allele can lead to Li-Fraumeni syndrome, characterized by development of several types of cancers [111]. In animal models, p53-/- mice develop cancers (mainly lymphomas and sarcomas) with nearly 100% penetrance [112]. Several proteins regulate p53, of which USP10 (ubiquitin carboxyl-terminal hydrolase protein-10) is one of the most important. By removing conjugated ubiquitin from target proteins, including p53, USP10 increases p53 stability in unstressed cells. This process is very important during DNA-damage response, in which USP10 translocates to the nucleus and deubiquitinates p53, stabilizing it and thus regulating its response to DNA damage [113,114,115]. It is thus clear that the reduction in USP10 activity caused by mycoplasma DnaK can have profound negative effects on the anti-cancer functions of p53.

**Figure 1:**
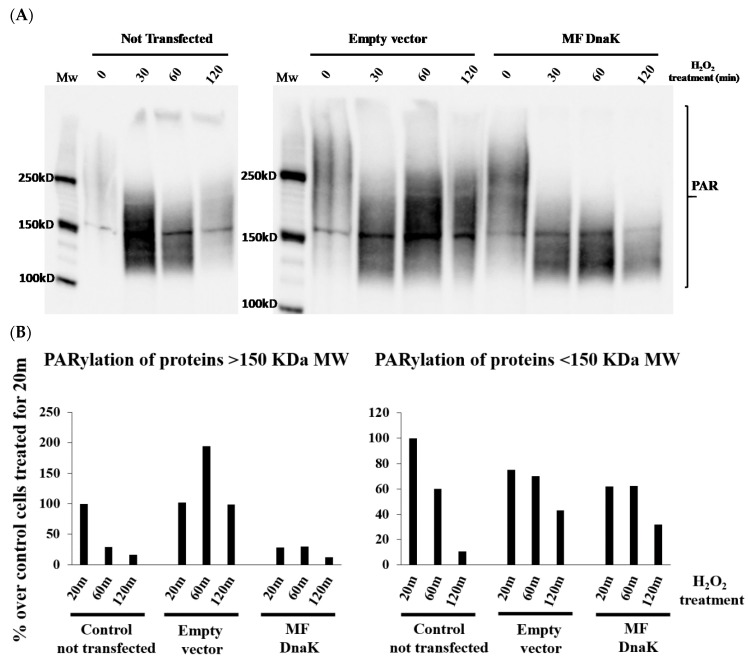
DnaK reduces PARylation in HCT116 cells. Following transfection, HCT116 cells were treated with H_2_O_2_ and collected at the indicated time points. Cells lysates were then subjected to SDS-PAGE 6% and protein parylation was detected by Western blot, using a specific anti-PAR antibody. MW are indicated. (**A**). Western blot analysis of HCT-116 cells. (**B**). Quantification of PARylated proteins.

In addition, we show that mycoplasma DnaK reduced PARylation activity of PARP1 following DNA damage (Figure 1). PARP 1 is one of the most studied members of the family of PARP proteins [116]. PARP1 is involved in the recognition and subsequent repair of single and double-strand breaks in DNA [117,118,119]. Following interaction with forms of damaged DNA, PARP1 activity is increased dramatically, resulting in PARylation of several proteins, including itself, histones, topoisomerase 1 (TOP1), DNA-dependent protein kinase (DNA-PK) and others [120], and in recruitment of single-strand break repair (SSBR)/base-excision repair (BER) factors to the damaged site [121]. Failure to properly repair DNA damage usually results in apoptosis, thus avoiding accumulation of DNA damage that could ultimately lead to cellular transformation. Mice lacking PARP1 exhibit high levels of sister chromatid exchange [122,123], increased chromosomal aberrations, including fusions, breaks, and telomere shortening [124], and double-mutant DNA-PK/PARP1-deficient mice develop a high frequency of T-cell lymphomas [125]. Following transfection with an expression vector carrying MF-I1 DnaK under the CMV promoter (see also [107], cells were treated at different time-points with H_2_O_2_, which causes DNA damage and promotes an increase in PARP activity. Protein PARylation appears as a smear of variable intensity, which in turn depends on the activity of PARP. We measured PARylation of proteins >150 KDa molecular weight (MW), and between 100 and 150 KDa and we observed two distinct patterns of PARylation in transfected cells. As represented in Fig 1B (left), PARylation of proteins >150 KDa in the control, the not-transfected cells reached a peak at 20 min post-treatment, and then rapidly decreased. This peak was high and delayed in cells transfected with the empty vector, indicating an effect of the transfection procedure, of the vector, or both. However, in the presence of the vector expressing MF-DnaK, PARylation of proteins >150 KDa MW was effectively abrogated (Figure 1B left). We observed a less pronounced difference in the PARylation of proteins between 100-150 KDa in cells transfected with the empty vector compared with the vector expressing MF-I1 DnaK (Figure 1B, right). Our data would indicate that the presence of DnaK greatly reduces the PARylation of certain proteins of very high MW (>150 KDa), while it seems to only marginally affect the PARylation of proteins between 100–150 KDa. This WB-based assay did not allow us to measure the PARylation of proteins <100 KDa, even though we previously demonstrated a reduction in histones PARylation in an in vitro assay [107]. We are currently investigating the underlying molecular mechanisms of this reduction and its relevance in vivo.

It should be noted that there are about 10^6^ PARP1 molecules [126,127,128] and about 10^4^ molecules of p53 per eukaryotic cell (http://book.bionumbers.org/what-are-the-concentrations-of-cytoskeletal-molecules/). On the other hand, the number of DnaK molecules measured in a single bacterial microorganism varies from 4x10^4^ to 1x10^5^, relative to its stress-induced status [129]. It thus appears that, under the proper stress-induced conditions, a single bacterium is able to produce enough DnaK molecules to severely affect the number of PARP1 and p53 molecules available to properly perform their DNA-repair and anti-cancer functions. For this reason, we believe that our data obtained by transfection experiments recapitulate what may be happening in vivo, i.e., that in cells where the DnaK is present, PARP1 and p53 activities will be reduced, increasing the likelihood of DNA instability and consequent malignant transformation.

**Figure 2:**
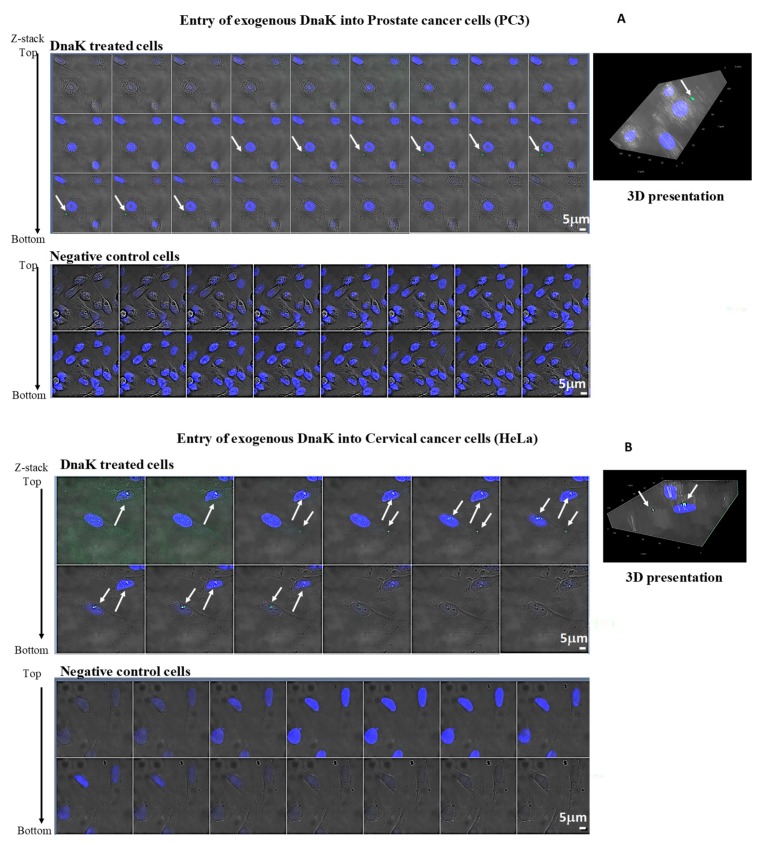
Intracellular uptake of exogenous DnaK-V5 by mycoplasma-free in PC-3 (**A**) and in HeLa cells (**B**). Confocal images of exogenous DnaK-V5 protein of M. fermentans in cells treated or not treated with DnaK-V5 protein. The figures show the collected Z-stacks of the corresponding gallery of images, each presenting a 0.5-µm-thick slide. A mouse monoclonal antibody, anti-V5, was used for primary labeling, and a FITC fluoresce-labeled polyclonal rabbit anti-mouse antibody was used for secondary labeling. Arrows indicates protein localization. Negative control cells, treated with primary and secondary antibodies alone without DnaK-V5 protein, are represented at the bottom of each figure. Insets in the upper right corners of A and B show a corresponding constructed 3D presentation of the protein uptake. Materials and Method for Figure 2, we followed the protocol described in [66] to perform the experiments described. Cell lines were obtained from the ATCC.

Bacteria can translocate proteins into eukaryotic cells either by attaching to the outside of the cellular membrane or by invading the cell [130,131]. In addition, prokaryotic and eukaryotic membrane-localized Hsp70 proteins may be released into the surrounding microenvironment and then translocate into the cytoplasm of nearby cells [132,133,134,135,136]. Using confocal microscopy, we were able to visualize and demonstrate that bystander, uninfected cells take up exogenous DnaK, suggesting a possible paracrine function in promoting cellular transformation, over and above direct mycoplasma infection (Figure 2A, B). Insets present a 3D assembly of the exogenous DnaK uptake by PC3 and HeLa cells (Figure 2A,B). Through immunoprecipitation studies, we previously demonstrated that mycoplasma DnaK is able to bind human DNAJ1A1 [107]. This could indeed indicate that, once in the intracellular compartments—either because released by invading bacteria or taken up by uninfected cells—some bacterial DnaKs may become functionally active by binding to the cellular co-chaperone DNAJA1. It is not clear at the moment what may be the extent of this exploitation, i.e., whether other DnaKs have the same ability, or whether other co-chaperones may be involved.

**Figure 3:**
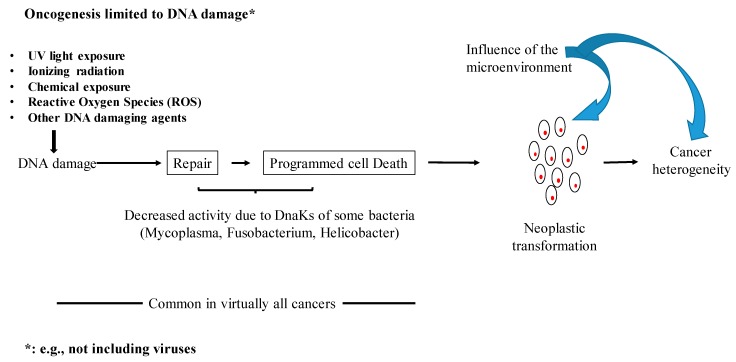
Some bacterial DnaKs may promote cellular transformation by affecting important cellular pathways.

Phylogenetic analysis showed that certain mycoplasmas, H. *pylori* and F. *nucleatum* have closely related DnaKs [107]. Definitive establishment of the causal correlation between *H. pylori* and gastric cancer provided the first demonstration that bacteria can cause cancer [137], and recent examples of studies in human patients highlighted an association between *F. nucleatum* and colorectal cancer [138,139,140,141,142,143,144]. Based on our data, it is tempting to speculate that a potential common mechanism could be involved in cellular transformation, where the DnaK of certain bacteria would interfere with pathways responsible for DNA repair and programmed cell death, with the consequent accumulation of mutations and a greatly increased chance of cellular transformation (Figure 3). However, additional experiments are still needed to support this hypothesis.

## 6. Conclusions

Several different bacteria have been recently associated with the origin of some human cancers, particularly those capable of establishing persistent intracellular infection, affecting host cell cycles and apoptotic pathways, and stimulating the production of inflammatory substances linked to DNA damage, thus potentially promoting DNA mutations and abnormal cell growth. The specific mechanism(s) whereby bacteria transform host cells are poorly understood and precise pathogen-cancer relationships remain largely elusive, except in the case of H. *pylori*, where a direct causal link has been demonstrated and a p53-related molecular mechanism implicated in cellular transformation has been described [137,145,146]. Based on our data, we propose that mycoplasmas—and perhaps certain other bacteria with closely related DnaK sequences and structure—have oncogenic potential mediated through the DnaK-dependent inhibition of DNA repair mechanisms and p53 function. DnaK could act in concert with some external factors—like smoking—previously shown to hamper the p53-p21 axis-dependent pathways [147]. The presence of some DnaKs would thus prevent the proper coordinated response that the cell engages to repair the DNA lesion following damage. This failure would allow the mutation to be fixed and transmitted along the cellular progeny. After a number of mutations occur, the cell would then transform. Mycoplasmas and certain other bacteria with similar DnaK may then be involved in the initiation of some cancers but not necessarily involved nor necessarily even be present in later stages. It is clearly of biological interest and potential therapeutic relevance to verify these findings in broader studies to understand the physical basis and the mechanism(s) responsible for reduced activities and levels of these critical cellular pathways. These studies may ultimately provide new preventive, diagnostic and therapeutic opportunities.

## 7. Materials and Methods

### 7.1. Cloning of *M. fermentans* MF-I1 DnaK.

The DnaK gene was synthesized at a commercial facility (Blue Heron Biotech, Bothell, WA). The nucleotide sequence was deduced from the genome sequence of M. *fermentans*. (MF-I1 strain) (ATFG00000000). Two tryptophan codons in the Mycoplasma sequence that are read as stop codon in eukaryotic cells were replaced by two tryptophan-codifying codons (G->A in positions 613 and 634). The TAG stop codon of the DnaK gene was deleted to make a fusion protein with the V5-(His)5 tag in the vector and cloned into the pcDNA 3.1 Directional/V5-His TOPO vector. After selection, clones were screened for the presence of the DnaK insert and the insert was sequenced to verify proper nucleotide composition [107]. DnaK is under the CMV promoter and it is expressed in HCT116 cells [107].

### 7.2. Expression and Purification of *M. fermentans* MF-I1 DnaK

Recombinant DnaK-V5 was obtained as previously described [107] briefly, MF-I1 DnaK sequence was inserted into a cloning vector, followed by the transformation and expression of the protein, subculture into TB/LB with Kanamycin, fractionation and purification (Biomatik USA, Wilmington, DE). After purification, the protein was extensively dialyzed against PBS1X, pH 7.4. Coomassie blue-stained SDS-PAGE (>85%) was used to determine purity. Aliquots of the protein were kept at −80 °C after reconstitution. Particular care was taken to avoid frequent freeze-thaws.

### 7.3. Transfection Assay

HCT116 cells (ATCC) were plated in 6-well plates at concentrations of 150,000 cells/well. After 24 h, cells were transfected with an expression vector containing M. *fermentans* MF-I1 DnaK. After about 32 h, cells were treated with H_2_O_2_ (750 μM) for the indicated time. Cells were then harvested, lysed in cell lysis buffer in the presence of protease inhibitors, subjected to SDS-PAGE 6%, and analyzed by Western blot. Anti-PAR monoclonal antibody (10H) was from Millipore Sigma. MW are indicated.

### 7.4. Quantification of Protein PARylation

Following developing with BioRad (ChemiDoc Imaging System), digital images were analyzed with ImageJ for pixel quantitation representing the PARylation of proteins >150 KDa or between 100–150 KDa. The data are expressed as percentage of control, not transfected cells treated for 20 min.

### 7.5. Confocal Microscopy Assay

Cells (PC3 or HeLA) were treated with DnaK-V5 protein. A mouse monoclonal antibody, anti-V5, was used for primary labeling, and a FITC fluoresce-labeled antibody was used for secondary labeling. Control samples have been stained with the same conditions described above in the absence of DnaK-V5. Confocal images of cell-associated fluorescence were acquired on Zeiss LSM800 confocal system (Carl Zeiss Microscopy, Germany). Zen Blue software was used to generate original images and Z-stacking option (1-micron size of sample slices) was utilized in order to achieve better information about mapping the protein areas of interest in three dimensions. All the parameters used in fluorescence microscopy were consistent in each experiment, including the laser excitation power, detector and offset gain.
